# Impact of Medication Adherence on Mortality and Cardiovascular Morbidity: Protocol for a Population-Based Cohort Study

**DOI:** 10.2196/resprot.8121

**Published:** 2018-03-09

**Authors:** Maria Giner-Soriano, Gerard Sotorra Figuerola, Jordi Cortés, Helena Pera Pujadas, Ana Garcia-Sangenis, Rosa Morros

**Affiliations:** ^1^ Medicines Research Unit Institut Universitari d’Investigació en Atenció Primària Jordi Gol Barcelona Spain; ^2^ Departament de Farmacologia, Terapèutica i Toxicologia Universitat Autònoma de Barcelona Bellaterra Spain; ^3^ Institut Català de la Salut Barcelona Spain; ^4^ Universitat Autònoma de Barcelona Bellaterra Spain; ^5^ Departament d'Estadística i Investigació Operativa Universitat Politècnica de Catalunya Barcelona Spain; ^6^ Spanish Clinical Research Network Unidad de Investigación Clínica y Ensayos Clínicos Institut Universitari d'Investigació en Atenció Primària Barcelona Spain

**Keywords:** cardiovascular diseases, coronary heart disease, acute coronary syndrome, adherence, aspirin, statins, beta-blockers, angiotensin-converting enzyme inhibitors, angiotensin-receptor blockers

## Abstract

**Background:**

Cardiovascular disease (CVD) is a group of disorders of the heart and blood vessels, such as coronary heart disease (CHD), cerebrovascular disease, and peripheral artery disease. CVD is the leading threat to global health, whether measured by mortality, morbidity, or economic cost. Long-term administration of aspirin, statins, beta-blockers, and angiotensin-converting enzyme inhibitors or angiotensin-receptor blockers improves survival in patients with stablished coronary heart disease. Nevertheless, adherence to prescribed medication is poor for long-term drug treatment.

**Objective:**

We aim to assess the relationship between adherences to the four pharmacological groups recommended for secondary prevention and the clinical outcomes of cardiovascular morbidity and mortality in patients with established CHD according to the level of adherence to these drugs in a population of incident cases of acute coronary syndrome (ACS).

**Methods:**

Population-based cohort study of patients with a first episode of ACS during 2006-2015 in the Information System for Research in Primary Care (SIDIAP) database. We will estimate adherence to these drugs. The primary endpoint is a composite of all-cause mortality, ACS, and ischaemic stroke. Bivariate analyses will be performed estimating odds ratios for categorical variables and mean differences for continuous variables. Hazard ratios for adherences will be calculated for outcome events using Cox proportional hazard regression models, and proportionality of hazards assumption will be tested.

**Results:**

We expect to estimate adherence to all four study treatments, the incidence of MACE, and to analyze if this incidence is associated with the level of drug adherence.

**Conclusions:**

We expect to find that adherent patients have a lower risk of the primary endpoints compared with nonadherent patients.

**Trial Registration:**

This study protocol was classified as EPA-OD by the AEMPS (IJG-EST-2017-01-2017-01, 07/04/2017) and registered in the EU PAS register (EUPAS19017, 09/05/2017).

## Introduction

Cardiovascular disease (CVD) is a group of disorders of the heart and blood vessels, such as coronary heart disease (CHD), cerebrovascular disease, and peripheral artery disease. CVD is the leading threat to global health, whether measured by mortality, morbidity, or economic cost [1]. In 2012, it was the leading cause of mortality worldwide, accounting for 31% of an estimated 56 million deaths from all causes. Also, CVD was responsible for the largest proportion of deaths for noncommunicable diseases under the age of 70 years, 37% of 16 million deaths [2].

Despite these numbers, the incidence of CVD death has decreased dramatically over the last four decades due to both population-level lifestyle changes in diet, smoking, and physical activity, and the development of effective interventions to treat individuals. The latter includes invasive procedures and effective drugs to tackle modifiable CVD risk factors [3].

A number of randomized clinical trials, meta-analyses and cohort studies have demonstrated that long-term administration of aspirin, statins, beta-blockers, and angiotensin-converting enzyme inhibitors (ACEI) or angiotensin-receptor blockers (ARB) improve survival in high-risk patients, particularly those with established CVD. Nevertheless, adherence to prescribed medication is poor for long-term drug treatment in CVD [1,4-6]. Different factors have been described to be related with long-term nonadherence [1,5-7].

In a recent cohort study conducted by Bansilal et al [4], 4015 patients who had suffered an acute myocardial infarction (AMI) were categorized according to their drug adherence to statin and ACEI into three categories: fully adherent (≥80% proportion of days covered [PDC]), partially adherent (40-79% PDC) or nonadherent (<40% PDC). Fully adherents had lower rates of major cardiovascular events (MACE) than partially adherents, 18.9% vs 24.7% (adjusted hazard ratio [HR] 0.81, 95% CI 0.69-0.94) and nonadherents, 18.9% vs 26.3% (HR 0.72, 95% CI 0.62-0.85).

In the cohort study conducted by Lafeber et al [8], 2706 CHD patients were included. Of them, 67% were treated with a combination of aspirin, statin, and at least one blood pressure (BP)-lowering agent for secondary prevention. After a median follow-up period of five years, the combination therapy compared with no combination showed lower rates for all events: AMI, HR 0.68 (95% CI 0.49-0.96); ischaemic stroke, HR 0.37 (95% CI 0.16-0.84); vascular mortality, HR 0.53 (95% CI 0.33-0.85); composite endpoint of the previous events, HR 0.66 (95% CI 0.49-0.88); and all-cause mortality, HR 0.69 (95% CI 0.49-0.96).

A population-based cohort study performed in Spain assessed adherence to secondary prevention drugs in a cohort of 7462 patients who survived an acute coronary syndrome (ACS) [6]. Medication adherence was evaluated by determining the PDC for each therapeutic group (antiplatelet agents, beta-blockers, ACEI or ARB, and statins) in the nine months following hospital discharge. Full adherence was defined as PDC75, at least 75% of days of the follow-up period covered by treatments dispensed. PDC75 for antiplatelet agents was reached by 5216 (69.9%) patients, for beta-blockers by 3231 (43.3%) patients, for ACEI/ARB by 3388 (45.4%) patients, and for statins by 4388 (58.8%). Only 3552 (47.6%) patients reached PDC75 for three or more therapeutic groups, whereas 1343 (18%) of patients did not reach PDC75 with any treatment. Some factors found to be related with nonadherence were older age, female sex, or copayment of drugs dispensed.

In a meta-analysis of 20 studies [9] in 376,162 patients assessing adherence to drugs for the primary or secondary prevention of a CHD event using prescription refill frequency, the estimated overall adherence to cardiovascular medications was only 57% (95% CI 50–64) after a median of 24 months, although it was superior in secondary prevention 66% (95% CI 56–75) than in primary prevention users (50%, 95% CI 45–56).

A large epidemiological study enrolled 7519 participants with established CVD from urban and rural communities in countries at various stages of economic development [10]. Use of antiplatelet drugs, beta-blockers, ACEI or ARB, and statins was assessed. Overall, 4421 (58.5%) individuals were not taking any of the four proven effective drugs, whereas 233 (3.1%) were taking all four drug types. Individuals recruited in high-income countries had had a CHD event or stroke a median of 6.0 years (interquartile range [IQR] 3.0–10.0) before inclusion. Although medication use increased in line with increase of country economic status, adherence rates in high-income countries were sparse too: 62.0% for antiplatelet drugs, 40.0% for beta-blockers, 49.8% for ACEI or ARB and 66.5% for statins.

A meta-analysis of randomised clinical trials assessed adherence to therapy comparing different dosing regimens in patients with chronic CVD.[11] The study showed that dosing regimens with once-daily administration, compared with two or more daily administrations, were associated with a significant 56% risk reduction of nonadherence to drug therapy (relative risk 0.44, 95% CI 0.35–0.54).

Due to the improvement of morbidity and mortality found with the quadruple drug therapy with antiplatelet, beta-blocker, ACEI or ARB, and statin in patients with established CVD, it is necessary to assess the long-term adherence to these drugs in the Catalan population and its relationship with cardiovascular events and mortality. Our hypothesis is patients with established CHD who adhere to drug therapy with the four recommended pharmacological groups have a lower risk of MACE and all-cause mortality compared with patients who do not adhere to drug therapy.

The main objective of our study is to assess the relationship between adherences to the four pharmacological groups recommended for secondary prevention and the clinical outcomes of cardiovascular morbidity and mortality in patients with established CHD. The outcomes which are included as components of the composite endpoint are all-cause mortality, ACS, and ischaemic stroke. The secondary objectives are: 1) to assess the incidence of the composite endpoint in patients who are adherent to treatment with all four drugs compared with patients who are adherent to any combination of three, two or one drug, or no drug; 2) to assess the relationship between baseline sociodemographic and clinical characteristics and adherence to drug therapy; 3) to compare the number of days on sickness leave due to any cause according to adherence to drug therapy; 4) to estimate prevalence of use of the four drug treatments; and 5) to describe the posology prescribed for the four drug treatments.

## Methods

### Study design

The study is a population-based retrospective cohort study.

### Study Period

Inclusion period was between 2006-2015. The follow-up period was up to 2016.

### Study Population

The study population includes individuals ≥18 years with an incident diagnosis of ACS during the study period 2006-2015, with at least two months of follow-up in the Information System for Research in Primary Care (SIDIAP) [12] after the index date. The next patients will be excluded: pregnant women on the index date; patients with a recorded diagnosis of ischaemic stroke in the six months prior to index date; patients living in a nursing home on the index date; and patients with Alzheimer’s disease or other dementias.

Case definition: patient with an incident diagnosis of ACS registered in CMBD-HA (dataset of diagnoses at hospital discharge) [13] of the Catalan Health Institute (ICS) within the period from 2006-2015. Index date definition: date of ACS episode.

### Data Collection and Data Sources

Diagnoses for study inclusion and endpoints will be obtained from CMBD-HA, which contains diagnoses at hospital discharge from all ICS hospitals, coded with International Classification of Diseases, Nineth Revision (ICD-9) [14]; see Table 1.

The rest of the variables will be captured from SIDIAP, which contains anonymized clinical information of all 279 PHC centres managed by the ICS in Catalonia (North-East Spain), covering a population of more than 5.8 million patients (about 80% of the total of 7.5 million population in Catalonia). The information contained in SIDIAP is registered by PHC general practitioners (GP), nurses and administrative staff in ECAP (electronic health records in ICS): comprehensive sociodemographic information, health conditions registered as ICD10 codes [15], specialist referrals, clinical parameters, toxic habits, PHC laboratory test results, GPs prescriptions and their corresponding pharmacy invoice data registered as Anatomical, therapeutic, chemical classification system (ATC) codes [16], date of sickness leave due to any cause, and date of death. Several reports have shown that SIDIAP data is useful for epidemiological research [17-25]. SIDIAP is listed under the European Network of Centres for Pharmacoepidemiology and Pharmacovigilance (ENCePP) resources database [26].

### Sample Size

The sample will be all patients with a first episode of ACS registered in CMBD-HA of ICS hospitals who meet all inclusion criteria and none of the exclusion criteria during the study period. In a previous study on patients with ACS conducted with SIDIAP database (publication pending) during the period 2009-2011, there were 3415 cases of ACS for all hospitals in Catalonia. Data from CMBD-HA of ICS hospitals corresponds approximately to 30% of all hospitals. Taking into account that our study period is 2006-2015 (10 years), we estimate to find approximately 3400 cases of ACS meeting inclusion criteria for our study.

### Variables

#### Exposure Definition

Patients will be classified as “exposed” to the study drugs (antiplatelet agents, beta-blockers, ACEI or ARB, statins) if they are prescribed any of them after the episode of ACS (up to two months after the event). The dose prescribed in ECAP will be considered the daily dose used for the patient, and the number of tablets contained in each package will cover the same number of days (see drugs of study in Table 2).

**Table 1:** International Classification of Diseases, Nineth Revision (ICD-9) codes for endpoints of study and procedures.

ICD-9 code	Description
411*	Unstable angina and other forms of acute coronary heart disease
410*	Acute myocardial infarction
433*, 434*, 435*, 436*, 437*	Ischaemic stroke
00.66, 36.03, 36.09, 39.50	Coronary angioplasty

**Table 2:** Anatomical, therapeutic, chemical classification system (ATC) codes for drugs of interest.

ATC code		Description of therapeutic group
**Study drugs**		
	B01AC	Platelet-aggregation inhibitors
	C07	Beta-blockers
	C09A, C09B	Angiotensin-converting enzyme inhibitors
	C09C, C09D	Angiotensin-receptor blockers
	C10AA, C10B	Statins
**Concomitant drugs**		
	C03	Diuretics
	C02	Antihypertensive drugs
	C08CA, C08D	Calcium-channel blockers (dihydropyridines/verapamil, diltiazem)
	B01AA, B01AB, B01AD, B01AE, B01AF, B01AX	Anticoagulants
	A10	Drugs used in diabetes mellitus
	C10AB, C10AC, C10AD, C10AX	Other lipid-lowering drugs
	C01A, C01B	Digoxin and antiarrhythmic drugs
	C01DA	Nitrates
	N05A	Antipsychotics
	M01A, N02BA, N02BB	Non-steroidal anti-inflammatory drugs

#### Adherence Definition

To estimate medication adherence, we will calculate the PDC for all four study treatments during eight months of follow-up after the index date. The PDC calculation is based on the packages dispensed and days of supply for each package, considering that the number of tablets contained in one package covers the treatment necessary for 28 or 30 days, depending on the drug. The information will be obtained from the pharmacy invoice data. For the PDC calculation, the numerator is the number of packages dispensed (invoice register) during the first 8 months of follow-up, and the denominator is the period of 8 months, which is the period for the adherence measure. Based on the PDC, patient adherence to each study drug is usually classified into one of two categories using the standard threshold of 75% (≥75%: adherent, <75%: nonadherent) [6,9]. PDC=75% accounts for six packages (each one including one month of drug treatment) dispensed during eight months. We define adherent patients as those who have received at least six packages during the first eight months after the event. Finally, according to adherence to all four study drugs, patients will be classified as adherent if they get the refill for all study drugs: PDC antiplatelet ≥75% + PDC beta-blockers ≥75% + PDC ACEI/ARB ≥75% + PDC statin ≥75%.

### Study Endpoints

ICD-9 codes for primary and secondary endpoints can be seen in Table 1. They will be captured from CMBD-HA database.

#### Primary Endpoint

The primary endpoint will be a composite endpoint of all-cause mortality, ACS and ischaemic stroke. From the index date (first episode of ACS), patients will be followed up to the end of follow-up or until a new diagnosis of any of the endpoints stated above. Patients who experience more than one endpoint during the study follow-up will be censored upon the first event of interest. Patients who do not experience any of the clinical events included in the composite endpoint during the follow-up will be censored at the last date of follow-up.

#### Secondary Endpoints

The secondary endpoints will be AMI, unstable angina, ischaemic stroke, all-cause mortality, overall number of days on sickness leave due to any cause and due to CVD events, prevalence of use of the four pharmacological groups of interest, posology of the four pharmacological groups of interest.

### Other Variables

All the following variables will be considered as potential confounders or effect modifiers in the association between adherence to the drug therapy and risk of the composite endpoint. They will be captured from SIDIAP database:

#### Patient Baseline Characteristics

All sociodemographic characteristics will be measured on the index date: index year, number of visits to PHC, age, sex, MEDEA index (socioeconomic deprivation index) [27], smoking status, alcohol intake, height, weight, Body Mass Index (BMI); the information comes primarily from a codified variable. If the patient has no information, it is calculated from height and weight and physical activity.

**Table 3:** ICD-10 codes for comorbidities of interest or diseases for exclusion

ICD-10 code	Description
I24*, I25*	Coronary heart disease
I63*, I65*, I66*, I67.2, I67.8	Ischaemic stroke
G45	Transient cerebral ischaemic attack
I70*, I73*, I74*	Peripheral vascular disease
E78*	Dyslipidaemia
I10*, I15*	Hypertension
E10*, E11*	Diabetes mellitus
I48	Atrial fibrillation
I50*	Heart failure
C00*-C97*	Malignancies
J40*-J44*	Chronic obstructive pulmonary disease
F30*-F39*	Depression
M05*, M06*, M15*-M19*	Arthritis (osteoarthritis or rheumatoid arthritis)
M80*, M81*	Osteoporosis
N18*	Chronic kidney disease
B20*-B24*	HIV
G30*, G31*	Alzheimer’s disease, other dementias

#### Comorbidities and Clinical Parameters

They will be measured closest to the index date: type of cardiovascular event at index date (AMI and unstable angina and other forms of ACS captured from CMBD-HA), presence of coronary angioplasty implant after the event (data source CMBD-HA), cholesterol and other lipid parameters (low density lipoprotein-cholesterol, high density lipoprotein-cholesterol, total-cholesterol, and triglycerides), blood pressure measured (systolic and diastolic blood pressure), glycated hemoglobin, glomerular filtration rate, serum creatinine, specific comorbid conditions (see ICD-10 codes in Table 3), Charlson comorbidity index [28,29].

#### Concomitant Drug Use

For all patients, baseline information on other medications for CVD prescribed throughout follow-up will be captured from the pharmacy invoice (see ATC codes for drugs in Table 2).

### Statistical analysis

Demographic and baseline characteristics of the participants will be described using frequencies and percentages for categorical variables and mean, standard deviation or median and interquartile range for continuous variables, as appropriate. Bivariate analyses will be performed estimating odds ratios for categorical variables and mean differences for continuous variables as well as their respective 95% CI. Multiple imputations by chained equations will be used to replace baseline missing values. Case-complete and imputed data results will be compared as a sensitivity analysis. The raw and adjusted HRs for adherences will be calculated for outcome events using Cox proportional hazard regression models, and proportionality of hazards assumption will be tested. Association analyses between adherence to study drugs, incidence of the endpoints or sick leave, and drug therapy (objectives 1, 2 and 3) will be analysed by means of generalized linear models. Objectives 4 and 5 are descriptive and they will be described using frequencies and percentages as appropriate.

### Ethical Aspects and Data Confidentiality

The present study follows national and international regulations: Declaration of Helsinki Ethical Principles for Medical Research Involving Human Subjects and Good Research Practice principles and guidelines. The study protocol has been approved by Institut Universitari d’Investigació en Atenció Primària (IDIAP) Jordi Gol Clinical Research Ethics Committee, the reference institution for research in PHC of the ICS, at May 3, 2017. Regarding the data contained in the databases and according to Spanish legislation about confidentiality and data protection (Ley Orgánica 15/1999 de 13 de diciembre de Protección de Datos de Carácter Personal), data included in SIDIAP are always anonymized. Thus, it is not necessary to ask for informed consent from the participants.

## Results

We expect to estimate adherence to all four study treatments, the incidence of MACE, and to analyze if this incidence is associated with the level of drug adherence. Adherence to drug treatment has shown better results in terms of risk reduction of MACE, so we expect to find that adherent patients have a lower risk of the primary endpoints in comparison with nonadherent patients.

## Discussion

We expect to find that adherent patients have a lower risk of the primary endpoints in comparison with nonadherent patients.

Selection bias is a common limitation in observational studies. In order to avoid this bias, where the population with missing data differs from those with complete data, missing values for continuous variables will be imputed instead of excluding records with missing data.

Another limitation is the presence of potential confounders. To minimize confounders’ effects, Cox regression models adjusted for sociodemographic characteristics and for possible confounders and predictive factors will be used.

## Abbreviations

ACEI: angiotensin-converting enzyme inhibitors
ACS: acute coronary syndrome
AEMPS: agencia Española de medicamentos y productos sanitarios
AMI: acute myocardial infarction
ARB: angiotensin-receptor blockers
ATC: anatomical, therapeutic, chemical classification system
BMI: body mass index
BP: blood pressure
CHD: coronary heart disease
CMBD-HA: conjunt mínim bàsic de dades a d’hospitalizació d’aguts (minimum dataset of
CVD: cardiovascular disease
ECAP: electronic health records in PHC
ENCePP: European Network of Centres for Pharmacoepidemiology and Pharmacovigilance
GP: general practitioner
HR: hazard ratio
ICD: International classification of diseases
ICS: Catalan Health Institute (Institut Català de la Salut)
IDIAP: Institut Universitari d’Investigació en Atenció Primària
IQR: interquartile range
MACE: major cardiovascular events
PDC: proportion of days covered
PHC: primary healthcare
SIDIAP: Information System for the Improvement of Research in Primary Care
